# The Knowledge–Practice Gap in Pharmacists’ Management of Suspected Meningococcal Disease During Mass Gatherings: Evidence from Surveys and Simulated Patient Visits

**DOI:** 10.3390/healthcare14213765

**Published:** 2026-11-04

**Authors:** Joud H. Alzahrani, Rimas M. Khayat, Lamar M. Abutaleb, Danah Y. Alhuthali, Atheer D. Alsaedi, Joud K. Alharbi, Abdulqader M. Abutaleb, Talal J. Qadah, Adel J. Dawood, Bashaer A. Alsharif, Saad M. Wali

**Affiliations:** 1 College of Pharmacy, Umm Al-Qura University, Makkah 24381, Saudi Arabia; 2 Pharmaceutical Sciences Department, College of Pharmacy, Umm Al-Qura University, Makkah 24381, Saudi Arabia; 3 Pharmacology and Toxicology Department, College of Pharmacy, Umm Al-Qura University, Makkah 24381, Saudi Arabia; smwali@uqu.edu.sa

**Keywords:** meningococcal disease, mass gatherings, community pharmacists, simulated patient, post-exposure prevention, referral practices, Hajj and Umrah, Saudi Arabia

## Abstract

**Background:** Meningococcal disease remains a public health concern during mass gatherings such as Hajj and Umrah, where crowding, close contact, and travel may increase transmission risk. Although pharmacists play a role in prevention and early referral, existing studies have relied on self-reported assessments, which may not reflect actual practice. **Aim:** To assess pharmacists’ knowledge, attitudes, and self-reported practices regarding the recognition, referral, and preventive management of suspected meningococcal disease during mass gatherings, and to compare reported practices with community pharmacists’ behavior observed during simulated patient encounters at the group level. **Methods:** A cross-sectional study was conducted from February to June 2026 in Makkah, Jeddah, Madinah, and Taif. A total of 380 community and hospital pharmacists completed a questionnaire assessing knowledge, attitudes, and self-reported practices regarding post-exposure meningococcal prevention. In parallel, 305 simulated patient visits assessed practice among community pharmacists using a standardized scenario. Spearman correlation, multivariable linear regression with robust standard errors, and chi-squared or Fisher’s exact tests (with Holm adjustment for regional comparisons) were used for analysis. **Results:** Pharmacists demonstrated good knowledge (median score, 5.0/6), attitudes (median, 13.0/15), and favorable self-reported practices (median, 15.5/20). However, only 5.2% of simulated patient encounters resulted in recognition of meningococcal disease, 7.5% included enquiry about close contact, and 6.2% included preventive counselling. Immediate referral was observed in 30.5% of encounters, while 88.9% involved symptomatic medication provision. Knowledge, attitudes, and self-reported practices were positively correlated with one another (Spearman’s rho = 0.32–0.54). **Conclusions:** A substantial group-level gap was observed between favorable self-reported practices and lower performance in a separate, unlinked sample of community pharmacists. Practical, case-based education, standardized referral pathways, and simulation-based training may help improve pharmacists’ ability to recognize and manage meningococcal disease during mass gatherings.

## 1. Introduction

Meningococcal disease is an acute, potentially fatal infection caused by *Neisseria meningitidis*, a Gram-negative diplococcus that can result in meningitis and septicemia, often with rapid clinical deterioration. The disease often presents with sudden onset of fever, headache, neck stiffness, and altered consciousness, and may rapidly progress to septic shock and death if untreated. Despite advances in antimicrobial therapy and vaccination, meningococcal disease remains associated with substantial morbidity and mortality worldwide [1].

Globally, the incidence of invasive meningococcal disease varies by region and serogroup, with an estimated case-fatality rate of 10–15% even with appropriate treatment, and up to 20% of survivors experiencing long-term complications such as neurological impairment or limb loss [1,2]. Periodic outbreaks continue to occur, particularly in settings characterized by crowding and close contact [2]. Transmission occurs via respiratory droplets, and asymptomatic nasopharyngeal carriage plays a critical role in disease spread [2].

Mass gatherings are defined as events in which the number of attendees is sufficient to strain the planning and response resources of the host community [3]. Such settings facilitate transmission because of crowd density, international travel, and prolonged close contact [3]. The Hajj and Umrah pilgrimages in Saudi Arabia represent some of the largest recurring mass gatherings globally, attracting millions of pilgrims annually [4]. Historically, the Hajj has been associated with meningococcal outbreaks, prompting the implementation of mandatory meningococcal vaccination policies and enhanced surveillance measures [4]. In this context, community pharmacists serve as highly accessible healthcare professionals who provide vaccination guidance, identify warning signs, and offer preventive counselling [5].

Several studies have explored meningococcal disease and carriage in the context of mass gathering settings. A systematic review and meta-analysis reported notable rates of *N. meningitidis* carriage among pilgrims, highlighting continued transmission risks despite vaccination requirements [6]. Similarly, a broader systematic review on mass gatherings and associated travel confirmed increased risk of meningococcal disease or carriage in such contexts [7]. The historical link between Hajj and international spread of meningococcal strains has been emphasized, reinforcing the importance of vaccination and preventive strategies [4].

Regarding pharmacy practice, a cross-sectional study demonstrated generally adequate knowledge among community pharmacists regarding common infectious illnesses and required vaccines during Hajj and Umrah, with high awareness regarding meningococcal vaccination [5]. However, simulated patient research suggests variability between reported knowledge and actual counselling performance. The concept of real-world behavior—capturing observed practice rather than self-reported responses—has emerged as a valuable complementary perspective in evaluating pharmacy services [8].

Simulated patient assessments offer a complementary means of evaluating counselling and clinical decision-making during standardized, unannounced encounters, helping to identify behaviors that may not be captured by self-reported questionnaires [9,10,11].

Although existing studies have examined meningococcal disease epidemiology during mass gatherings and assessed general pharmacists’ knowledge and perceptions during Hajj and Umrah, evidence remains limited on how pharmacists recognize and manage presentations suggestive of meningococcal disease in these settings. Moreover, most available data rely on self-reported surveys, which may not fully capture behavioral patterns in actual practice environments. There is a need for research that addresses both the topic-specific gap and the methodological limitations of prior work. Accordingly, this study aimed to assess pharmacists’ knowledge, attitudes, and self-reported practices regarding the recognition, referral, and preventive management of suspected meningococcal disease during mass gatherings, and to compare these findings at the group level with community pharmacists’ observed behavior during simulated patient encounters.

## 2. Materials and Methods

### 2.1. Study Design

A cross-sectional study with two complementary components was conducted concurrently between February and June 2026: a quantitative survey and an unannounced simulated patient (SP) assessment. The survey evaluated pharmacists’ knowledge, attitudes, and practices regarding post-exposure preventive counselling for meningococcal disease during mass gatherings, while the SP assessment evaluated real-world clinical performance. Combining these approaches enabled a more comprehensive assessment of pharmacy practice by comparing self-reported behavior with observed practice and by addressing the limitations of relying solely on self-reported data. The study was reported in accordance with the Strengthening the Reporting of Observational Studies in Epidemiology (STROBE) guidelines [12]. The completed STROBE checklist is provided as STROBE Checklist S3. The survey and simulated patient components involved separate, unlinked samples; comparisons between them were descriptive and at the group level, not paired comparisons of individual pharmacists.

### 2.2. Study Setting and Participants

The study was conducted in the Western Region of Saudi Arabia, encompassing the cities of Makkah, Jeddah, Madinah, and Taif. This region was selected due to its unique relevance to mass gatherings, particularly during the Hajj and Umrah seasons, which attracted millions of pilgrims from diverse geographical and cultural backgrounds. Licensed pharmacists practicing in community and hospital pharmacy settings were eligible for participation. Participants were recruited either electronically through an online survey or through direct, in-person recruitment at their respective pharmacy settings.

The study period overlapped with Ramadan (18 February–19 March 2026), a period of increased Umrah activity, and the Hajj pilgrimage season (principal rituals, 25–30 May 2026).

### 2.3. Survey Instrument

Data were collected using a structured, self-administered questionnaire developed to evaluate three primary domains: knowledge, attitudes, and practices regarding post-exposure preventive counselling for meningococcal disease (Questionnaire S1). The knowledge domain consisted of six items covering meningococcal disease etiology, transmission, vaccination efficacy, the necessity of post-exposure prevention, symptom recognition, and recommended management. The attitudes domain consisted of five 4-point Likert-type items ranging from 0 (Strongly disagree) to 3 (Strongly agree). The practices domain consisted of five 5-point Likert-type items ranging from 0 (Never, 0%) to 4 (Always, >75%). Demographic and professional characteristics were also collected, including age, gender, educational qualification, practice setting, city of practice, and years of professional experience. To assess the clarity and comprehensibility of the questionnaire, the instrument was pilot-tested among 15 pharmacists, comprising nine community pharmacists and six hospital pharmacists. Feedback from the pilot testing was used to identify ambiguous or unclear wording and to inform minor refinements before finalization of the questionnaire. The questionnaire items were developed to address gaps identified in previous studies. Prior to pilot testing, the questionnaire underwent a qualitative content review by a panel of five experts from healthcare-related disciplines, who evaluated the relevance and clarity of the items. The expert panel recommended no modifications, and no formal quantitative content validity index was calculated.

The questionnaire was administered as an open-book survey: participants were not prohibited from consulting external sources while responding. Survey participants were informed of the study title and meningococcal disease focus before providing consent.

### 2.4. Simulated Patient (SP) Visits

To evaluate community pharmacists’ practice under standardized real-world conditions, trained data collectors conducted simulated patient (SP) visits across community pharmacies in four target cities in Saudi Arabia (Makkah, Jeddah, Madinah, and Taif), with each pharmacy assessed only once to avoid repeated observations. A total of ten data collectors participated as SPs. All data collectors underwent standardized training prior to study implementation, which included orientation on the study objectives, familiarization with the simulated patient methodology, scenario scripts, expected response flow, and role-play rehearsals to maintain inter-evaluator consistency across visits.

The ten simulated patients were assigned to four cities: Jeddah (3 SPs; 85 visits), Makkah (3 SPs; 100 visits), Madinah (2 SPs; 60 visits), and Taif (2 SPs; 60 visits). Each SP conducted visits only in their assigned city. The cities were selected for their relevance to Hajj and Umrah; pharmacies were approached to achieve feasible geographical coverage across neighborhoods rather than through random sampling. All 305 visits were completed, with no refusals or incomplete encounters.

A single standardized clinical scenario depicting a patient with symptoms suggestive of meningococcal disease was used across all encounters. The use of a standardized scenario was intended to minimize inter-SP variability and enhance comparability of pharmacist responses across pharmacies, cities, and data collectors. To support content validity and clinical accuracy, the scenario script and standardized questions were reviewed and face-validated by researchers with expertise in clinical pharmacy.

Role-play rehearsals and a shared scenario were used for standardization, but formal inter-rater reliability was not measured. The SPs themselves completed the assessment checklists; no additional observers were present. Visits were unannounced, and pharmacists were not informed that the encounter was simulated.

During each visit, the simulated patient presented the standardized symptoms without explicitly stating a suspected diagnosis. Immediately following each encounter, the data collector completed a structured assessment form to document predefined process and management outcomes. The assessment captured whether the pharmacist recognized the possibility of meningococcal disease based on the presenting symptoms or, at minimum, suspected an underlying infection without specifically identifying meningococcal disease. It also assessed whether the pharmacist enquired about recent close contact with a suspected or confirmed meningococcal case, advised immediate referral to a healthcare facility, and provided advice on measures to prevent further transmission following suspected exposure. In addition, the assessment documented whether medications for symptomatic relief were provided and whether any inappropriate management was attributed to time pressure or high patient volume.

Specific meningococcal recognition was recorded when the pharmacist explicitly identified meningococcal disease as a possibility; general infection suspicion was recorded when infection was mentioned without naming meningococcal disease. The checklist separately recorded whether the pharmacist asked about close contact, recommended urgent referral, gave preventive advice, or offered symptomatic medication. These were encounter-level observations, not assessments of chemoprophylaxis for asymptomatic contacts. No medications were purchased, and individual medication names were not recorded. The standardized simulated patient script and post-visit assessment checklist are provided in Supplementary Materials S2.

### 2.5. Sample Size Calculation

Sample size was calculated using G*Power 3.1.9.7 for multivariable linear regression with fixed model and R^2^ deviation from zero. Assuming a medium effect size (f^2^ = 0.15), alpha of 0.05, and statistical power of 0.95 with 14 predictor parameters (the dummy-coded categories of gender, age, educational qualification, practice setting, city, and years of experience), a minimum total sample size of 194 participants was required; the final sample of 380 participants exceeded this requirement.

For the simulated patient component, the sample size was calculated using the formula for estimating a single proportion, *n* = *Z*^2^*p*(1 − *p*)/*d*^2^. Assuming an expected proportion of appropriate pharmacist recognition of 14.9%, based on a previous simulated patient study conducted in the same region [13], a 95% confidence level (*Z* = 1.96), and a margin of error of 4%, the minimum required number of visits was 305.

### 2.6. Scoring

Knowledge was assessed using six items regarding meningococcal disease etiology, transmission, vaccination efficacy, post-exposure prevention necessity, symptom recognition, and recommended management. Each correct response was assigned 1 point and incorrect responses 0 points, yielding a total knowledge score ranging from 0 to 6. Attitudes were measured using five Likert-type items with response options scaled from 0 (Strongly disagree) to 3 (Strongly agree), generating a composite attitudes score ranging from 0 to 15. Practices were evaluated using five Likert-type items with response options ranging from 0 (Never, 0%) to 4 (Always, >75%), producing a composite practices score ranging from 0 to 20. Higher scores on all three domains reflected greater knowledge, more favorable attitudes, and more frequent implementation of post-exposure preventive counselling practices, respectively. Because the total knowledge score summarizes six heterogeneous items, a high total score may coexist with specific item-level gaps; the proportion of correct responses to each item was therefore also reported.

### 2.7. Statistical Analysis

Analyses were performed using R version 4.6.0 (R Foundation for Statistical Computing, Vienna, Austria) and RStudio 2026.05.1 Build 225 (Boston, MA, USA), with statistical significance defined as *p* < 0.05. Descriptive statistics were calculated for all demographic and professional characteristics, and internal consistency of the knowledge, attitudes, and practices domains was assessed using Cronbach’s alpha (with Feldt 95% confidence intervals) and McDonald’s omega; for the binary knowledge items, Cronbach’s alpha is equivalent to the Kuder–Richardson formula 20. Item-level analysis included the proportion of correct responses (knowledge items) or item means (attitude and practice items), corrected item–total correlations, and alpha if item deleted (Table S1). Univariable linear regression analyses examined associations between independent variables (gender, age, educational qualification, practice setting, city, years of experience) and each outcome (knowledge, attitudes, and practices scores). Separate multivariable linear regression models were then fitted for the knowledge, attitudes, and practices scores, each including all six sociodemographic and professional factors entered simultaneously. The domain scores were not entered as covariates in one another’s models, to avoid circular mutual adjustment; given the cross-sectional design, all estimates are interpreted as associations rather than predictive or causal effects. The bivariate interrelationships among the knowledge, attitudes, and practices scores were examined separately using Spearman’s rank correlation coefficients with bootstrap 95% confidence intervals (2000 resamples). Model assumptions were evaluated using the Breusch–Pagan test for homoscedasticity, the Shapiro–Wilk test for normality of residuals, generalized variance inflation factors for multicollinearity, and Cook’s distance (threshold 4/*n*) for influential observations; independence of observations was ensured by design, as each pharmacist contributed a single response. Because the residuals departed from normality and influential observations were present, all observations were retained in the primary analysis, and 95% confidence intervals and *p*-values were derived from heteroscedasticity-consistent (HC3) robust standard errors. Two sensitivity analyses were performed: nonparametric bootstrap percentile 95% confidence intervals (2000 case resamples) and refitting each model after excluding observations with Cook’s distance > 4/*n*. Simulated patient outcomes were summarized as frequencies and percentages with exact (Clopper–Pearson) 95% confidence intervals, which remain informative when no events are observed. Regional differences were assessed using Pearson’s chi-squared test or Fisher’s exact test, as appropriate. Because seven regional comparisons were performed, *p*-values were additionally adjusted for multiple testing using the Holm method, and the regional analyses were considered exploratory.

There were no missing values in the final analytic dataset; therefore, no imputation or missing-data exclusions were necessary. The Shapiro–Wilk test was used to assess normality of domain-score distributions; non-normal scores were summarized using medians and interquartile ranges.

### 2.8. Ethics Approval and Consent to Participate

Ethical approval was obtained from the Scientific Biomedical Research Ethics Committee at the College of Medicine, Umm Al-Qura University, Saudi Arabia (HAPO-02-K-012-2026-03-3300), and the study was conducted in accordance with the Declaration of Helsinki.

For covert simulated patient encounters, prior informed consent was not sought and no post-visit debriefing was conducted. No separate consent waiver was obtained. The encounters involved no medication purchases or administration, and the simulated patient procedure was included in the approved study protocol.

Participants were informed about the purpose of the study and assured that their responses would remain anonymous and confidential. Participation was voluntary, and digital written informed consent was obtained from all participants prior to participation.

## 3. Results

### 3.1. Demographic and Professional Characteristics

Among 380 pharmacists included in the current study, males constituted more than half of the sample (212, 55.8%), with the predominant age group being 23–28 years (250, 65.8%). The majority held a Bachelor of Pharmacy (PharmD) degree (216, 56.8%), worked in community pharmacy settings (206, 54.2%), and were based in Jeddah (117, 30.8%). Experience levels showed that 1–5 years was the most common duration (163, 42.9%, Table 1).

### 3.2. Knowledge About Meningococcal Disease and Post-Exposure Prevention

Most participants correctly identified *Neisseria meningitidis* as the causative organism (304, 80.0%), understood that close contact increases transmission risk (313, 82.4%), recognized that vaccination does not completely eliminate disease transmission (186, 48.9%), and acknowledged that post-exposure preventive counselling remains necessary among vaccinated individuals (315, 82.9%). The majority could identify the symptom cluster most suggestive of meningococcal disease requiring immediate referral (311, 81.8%) and recognized that immediate healthcare facility referral is the recommended action following confirmed meningococcal exposure (256, 67.4%, Table 2).

### 3.3. Attitudes Toward Post-Exposure Preventive Counselling

**Figure 1:**
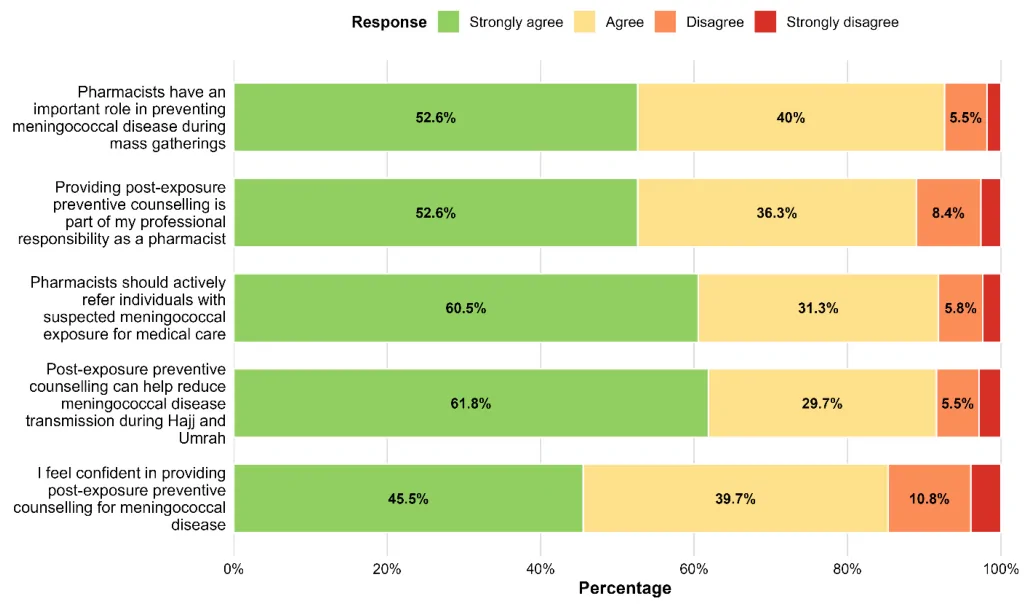
Participants’ responses to attitudes items, N = 380.

Strong agreement or agreement with the importance of the pharmacist’s role in preventing meningococcal disease during mass gatherings was reported by 352 participants (92.6%), and 338 (89.0%) agreed or strongly agreed that post-exposure preventive counselling is part of their professional responsibility. Regarding active referral for suspected exposure, 349 participants (91.8%) agreed or strongly agreed this is a pharmacist responsibility. The belief that post-exposure preventive counselling reduces transmission during Hajj and Umrah was endorsed by 348 participants (91.6%), while 324 (85.3%) felt confident in providing such counselling (Figure 1).

### 3.4. Self-Reported Practices During Mass Gatherings

**Figure 2:**
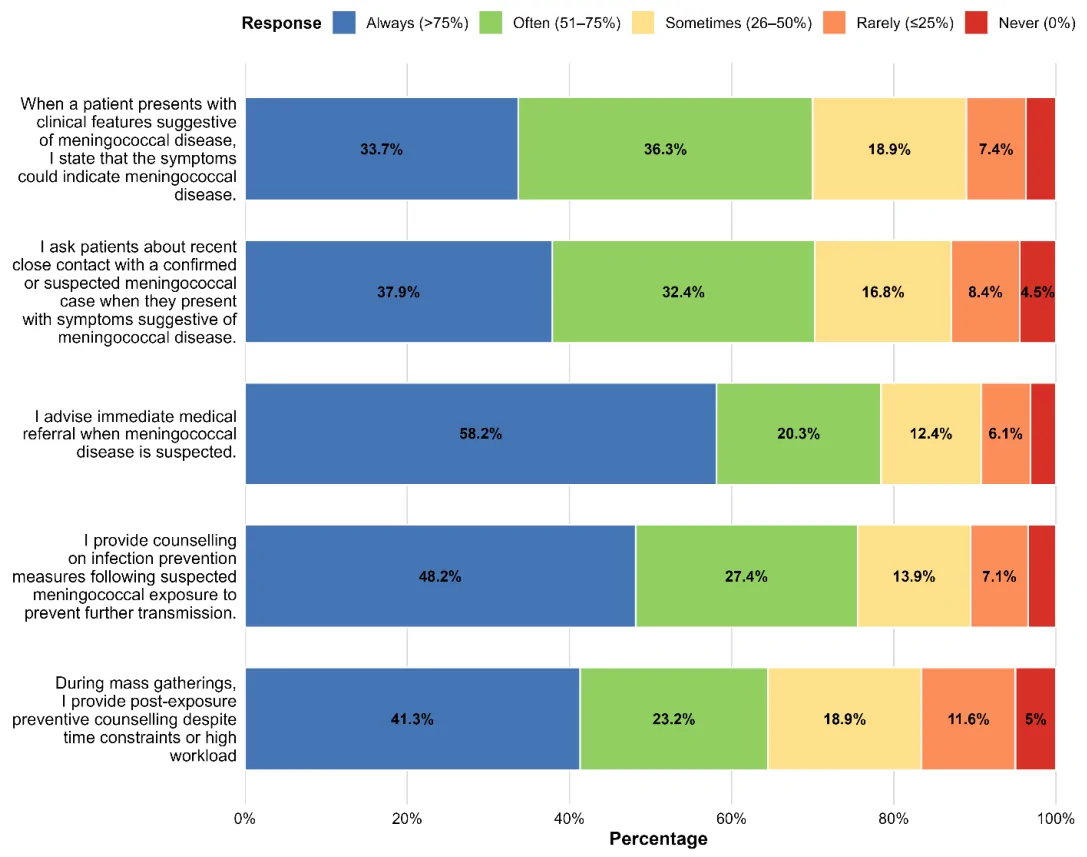
Participants’ responses to practice items, N = 380.

When patients presented with symptoms suggestive of meningococcal disease, 266 pharmacists (70.0%) reported often or always stating that the symptoms could indicate meningococcal disease. Regarding enquiry about recent close contact with suspected or confirmed meningococcal cases, 267 pharmacists (70.3%) reported doing so often or always. Most participants (298, 78.4%) reported often or always advising immediate medical referral when meningococcal disease was suspected. Infection prevention counselling following suspected exposure was provided often or always by 287 pharmacists (75.5%). Despite time constraints or high workload, 245 participants (64.5%) reported often or always providing post-exposure preventive counselling during mass gatherings (Figure 2).

### 3.5. Descriptive Statistics of the Domains

The knowledge domain (6 items) demonstrated a median score of 5.0 (IQR 4.0–6.0), with Cronbach’s alpha of 0.631 (95% CI 0.570–0.685; McDonald’s omega = 0.673). The attitudes domain (5 items) showed a median score of 13.0 (IQR 10.0–14.0), with Cronbach’s alpha of 0.756 (95% CI 0.715–0.793; omega = 0.758). The practices domain (5 items) had a median score of 15.5 (IQR 13.0–18.0), with Cronbach’s alpha of 0.809 (95% CI 0.777–0.838; omega = 0.812; Table 3). All three domain scores were non-normally distributed (Shapiro–Wilk *p* < 0.001) and are therefore summarized using medians and interquartile ranges.

Item-level analysis (Table S1) showed that the modest internal consistency of the knowledge domain was mainly attributable to two items: the vaccination item (correctly answered by 48.9%, 95% CI 43.8–54.1; corrected item–total correlation = 0.24) and the item on the recommended action after exposure (67.4%, 95% CI 62.4–72.1; corrected item–total correlation = 0.17). Deleting either item would have increased alpha only marginally (to 0.647 and 0.666, respectively); therefore, all six items were retained. Although the median knowledge score was 5 of 6, only 97 participants (25.5%) answered all six items correctly, and the vaccination item was the only item answered correctly by fewer than two-thirds of participants. Corrected item–total correlations ranged from 0.48 to 0.56 for the attitude items and from 0.48 to 0.67 for the practice items.

Knowledge, attitudes, and practices scores were positively correlated: knowledge and attitudes (Spearman’s rho = 0.36, 95% CI 0.27–0.44), knowledge and practices (rho = 0.32, 95% CI 0.22–0.40), and attitudes and practices (rho = 0.54, 95% CI 0.46–0.61; all *p* < 0.001).

### 3.6. Factors Associated with Knowledge

In the multivariable model including all six sociodemographic and professional factors (all 380 participants; HC3 robust standard errors), a doctoral (PhD) qualification was associated with lower knowledge scores compared with a Bachelor of Pharmacy (B.Pharm) degree (β = −1.55, 95% CI −2.76 to −0.34, *p* = 0.012), and practicing in Madinah was associated with higher knowledge scores compared with practicing in Makkah (β = 0.44, 95% CI 0.03 to 0.84, *p* = 0.035). No other factor was significantly associated with knowledge. The model explained 7.5% of the variance in knowledge scores (adjusted R^2^ = 3.9%; Table 4). Both associations were consistent in the bootstrap and influential-observation sensitivity analyses (Table S2), although the PhD subgroup was small (*n* = 15); 1–5 years of experience reached statistical significance only after the exclusion of influential observations.

### 3.7. Factors Associated with Attitudes

Female pharmacists had lower attitude scores than male pharmacists (β = −0.94, 95% CI −1.52 to −0.35, *p* = 0.002), and pharmacists with 1–5 years of experience had lower attitude scores than those with less than 1 year of experience (β = −0.70, 95% CI −1.35 to −0.05, *p* = 0.035). The model explained 6.3% of the variance in attitude scores (adjusted R^2^ = 2.7%; Table 5). Both associations were consistent in the sensitivity analyses; practicing in Madinah was associated with higher attitude scores only after the exclusion of influential observations (Table S2).

### 3.8. Factors Associated with Practices

Compared with pharmacists with less than 1 year of experience, those with 1–5 years (β = −1.54, 95% CI −2.61 to −0.47, *p* = 0.005) and 6–10 years of experience (β = −1.86, 95% CI −3.51 to −0.20, *p* = 0.028) reported lower practice scores. Female sex showed a borderline association with lower practice scores (β = −0.97, 95% CI −1.96 to 0.01, *p* = 0.053). The model explained 4.6% of the variance in practice scores (adjusted R^2^ = 1.0%; Table 6). The association with 1–5 years of experience was consistent in both sensitivity analyses, whereas the association with 6–10 years was supported by the bootstrap confidence interval (−3.52 to −0.16) but was attenuated after the exclusion of influential observations (*p* = 0.066; Table S2).

### 3.9. Simulated Patient Observations

Across 305 simulated patient visits, only 16 pharmacists (5.2%, 95% CI 3.0–8.4) recognized the possibility of meningococcal disease based on presented symptoms. In addition, only 92 pharmacists (30.2%, 95% CI 25.1–35.7) suspected an infection without specifically identifying meningococcal disease. Few pharmacists (23, 7.5%, 95% CI 4.8–11.1) enquired about recent close contact with a suspected or confirmed meningococcal case. Overall, 93 pharmacists (30.5%, 95% CI 25.4–36.0) advised immediate referral to a healthcare facility. Only 19 pharmacists (6.2%, 95% CI 3.8–9.6) provided advice on preventing further transmission following suspected exposure. Medications for symptomatic relief were provided by 271 pharmacists (88.9%, 95% CI 84.8–92.2), with regional variation evident. Inappropriate management attributed to time pressure or high patient volume was reported in 19 instances (6.2%, 95% CI 3.8–9.6, Table 7).

Considering regional variations (exploratory analyses), Taif demonstrated notably low performance across the key variables: pharmacists in Taif showed zero recognition of meningococcal disease possibility (0.0%) compared to 10.6% in Jeddah, 5.0% in Makkah, and 3.3% in Madinah (*p* = 0.034; Holm-adjusted *p* = 0.101). Similarly, no pharmacists in Taif suspected infection without meningococcal specification (0.0%), whereas Madinah and Jeddah showed substantially higher rates at 50.0% and 49.4%, respectively, compared to 20.0% in Makkah (*p* < 0.001; Holm-adjusted *p* < 0.001). Enquiry about recent close contact was absent in Taif (0.0%) but occurred in 14.1% of Jeddah visits, 8.0% in Makkah, and 5.0% in Madinah (*p* = 0.007; Holm-adjusted *p* = 0.036). Advice for immediate referral was not documented in any Taif visit (0.0%), contrasting sharply with Madinah (58.3%), Jeddah (50.6%), and Makkah (15.0%; *p* < 0.001; Holm-adjusted *p* < 0.001). Conversely, medication provision for symptomatic relief reached 100.0% in Taif, significantly higher than Jeddah (87.1%), Madinah (86.7%), and Makkah (85.0%; *p* = 0.022; Holm-adjusted *p* = 0.090, Table 7). For outcomes with no events in Taif (0 of 60 visits), the exact 95% CI was 0.0–6.0%. After Holm adjustment, regional differences remained statistically significant for suspected infection, enquiry about close contact, and referral advice, but not for recognition of meningococcal disease or provision of symptomatic medication.

## 4. Discussion

Overall, pharmacists demonstrated relatively good knowledge, positive attitudes, and favorable self-reported practices. However, the simulated patient assessment, which was conducted separately among community pharmacists, revealed substantially lower performance across several clinically important components of post-exposure management, including recognition of possible meningococcal disease, enquiry about recent close contact, provision of preventive advice, and referral to a healthcare facility. The principal finding was therefore a substantial group-level discrepancy between favorable self-reported practices and lower observed performance across independent, unlinked samples, rather than a within-pharmacist difference. This finding is particularly relevant in the context of Hajj and Umrah, where high population density, prolonged close contact, and international travel create conditions conducive to the transmission of infectious diseases [1,2,3,4,14].

### 4.1. Knowledge and Attitudes Toward Post-Exposure Prevention

Community pharmacists are among the most accessible healthcare professionals during mass gatherings, and adequate knowledge of meningococcal disease is a prerequisite for effective post-exposure counselling. In the present study, the overall knowledge score was acceptable, yet an important item-level deficiency was identified: whereas 80.0% of participants correctly identified *Neisseria meningitidis* as the causative organism, 82.4% recognized that close contact increases transmission risk, and 82.9% acknowledged that post-exposure counselling remains necessary among vaccinated individuals, only 48.9% recognized that vaccination does not completely eliminate the possibility of transmission. This pattern indicates that pharmacists possess sound factual awareness of the disease but demonstrated a specific knowledge gap regarding the extent of protection afforded by vaccination. These findings are broadly consistent with previous Saudi research: Alghadeer et al. reported generally adequate knowledge and favorable perceptions of Hajj- and Umrah-related conditions among community pharmacists, including awareness of vaccination requirements [5], and Alorfi et al. similarly documented favorable knowledge and attitudes in the Western Region [15]. The present study extends this literature by focusing specifically on post-exposure preventive counselling rather than vaccination awareness alone. This knowledge gap is nonetheless clinically relevant, because meningococcal carriage persists among pilgrims despite vaccination; systematic reviews and meta-analyses have continued to document nasopharyngeal carriage during Hajj and Umrah [6,7], carriage of 4.6% was reported in one pilgrim cohort [16], and serogroup W-135 carriage was detected among vaccinated [4,17]. Maintaining clinical vigilance therefore remains important when compatible symptoms or a relevant exposure history are present, even among vaccinated individuals.

Attitudes constitute a distinct construct from knowledge and are frequently proposed as the professional factors associated with behavior. In this study, attitudes were strongly favorable, with more than 90% of pharmacists agreeing or strongly agreeing that they have an important role in preventing meningococcal disease and that referral of suspected exposure is a professional responsibility. This is an important observation because it indicates that the deficiencies later revealed in observed practice cannot readily be attributed to a lack of recognition of the pharmacist’s professional role. The favorable attitudes align with the positive professional dispositions previously reported among Saudi community pharmacists [5,15]. By contrast, these strong attitudes did not translate into commensurate observed behavior, pointing to a meaningful distinction between recognizing the importance of a responsibility and consistently discharging that responsibility during a real encounter, a divergence that has also been reported in other pharmacy-practice settings when self-reported and observed practice are compared [8,18].

### 4.2. The Gap Between Self-Reported and Observed Practice

The most important finding of this study was the pronounced difference between what pharmacists reported doing and what they were observed to do. Approximately 70% of participants reported that they often or always recognized symptoms suggestive of meningococcal disease, yet only 5.2% did so during simulated patient encounters; 70.3% reported frequently asking about recent close contact, compared with 7.5% observed; preventive counselling was reported as being provided often or always by 75.5% of participants but was observed in only 6.2% of visits; and although 78.4% reported routinely advising immediate referral, referral was observed in only 30.5% of encounters. These findings should not be interpreted as evidence of intentional misreporting; rather, they indicate that self-reported instruments yield a substantially more favorable picture of practice than direct observation. This pattern is highly consistent with prior pharmacy-practice research, including research on counselling practices by Alaqeel and Abanmy in Riyadh [18] and simulated patient work by Al Qarni et al. in the Bisha directorate [8]. It also closely parallels a simulated patient study conducted in Western Saudi Arabia on drug–drug interaction management in the same region, in which only 14.9% of pharmacists recognized a clinically significant hazard in real time despite adequate survey-based knowledge, reinforcing that the knowledge–practice gap is not confined to a single clinical scenario [13]. A degree of caution is nonetheless warranted, because survey and simulated patient responses could not be linked at the individual level; the observed difference therefore represents a group-level discrepancy rather than proof that the same pharmacists who reported good practice subsequently failed to perform it, and the magnitude of such gaps varies across studies depending on scenario complexity and setting.

Several complementary frameworks help explain why this gap arises. From a cognitive perspective, the discrepancy is consistent with the well-established distinction between declarative knowledge (knowing that) and procedural knowledge (knowing how to apply it under real conditions). In pharmacy education, simulation-based approaches have been used to bridge the gap between knowledge acquisition and the application of clinical knowledge and skills in practice [19]. Self-administered questionnaires predominantly assess declarative knowledge, whereas an actual patient encounter requires the rapid integration of that knowledge under time pressure and cognitive load. In the high-volume environment characteristic of mass gatherings, pharmacists may default to heuristic, pattern-based processing that is efficient for routine requests but error-prone for low-frequency, high-stakes syndromes such as invasive meningococcal disease, a mechanism supported by studies of pharmacists’ clinical decision making and of clinicians’ cognitive management of complex interactions [20,21]. The consistency of this pattern across multiple settings also underscores the methodological value of covert simulated patient observation, which captures behavior under standardized, naturalistic conditions that self-report cannot directly assess; the approach is well validated in pharmacy-practice research and in the wider health-services literature [9,10,11,22,23]. The principal caveat is that a simulated patient visit captures a single standardized encounter and may not reflect a pharmacist’s consistent behavior across the full range of presentations encountered in routine practice.

### 4.3. Clinical Significance of the Observed Behavior

The low recognition rate is particularly important when viewed against the clinical tempo of meningococcal disease. Only 5.2% of pharmacists recognized the possibility of meningococcal disease, although a further 30.2% suspected an infection without identifying the specific syndrome. The latter finding suggests that many pharmacists perceived an abnormal or potentially infectious presentation but did not translate the clinical features into recognition of a specific high-risk condition. Because meningococcal disease can progress rapidly and may present initially with non-specific features before severe complications develop [1,2,24], pharmacists are not expected to establish a definitive diagnosis of meningococcal disease in the community pharmacy setting; however, recognition of compatible red-flag features should facilitate prompt referral to an appropriate healthcare facility. Interpreted in this light, the low rate of disease-specific recognition highlights an important area for improvement; however, failure to identify meningococcal disease specifically does not necessarily indicate inappropriate management, as urgent referral may be clinically appropriate even without a disease-specific diagnosis.

A parallel concern applies to exposure assessment, referral, and dispensing behavior. Enquiry about recent close contact was observed in only 7.5% of encounters, even though close contacts of a confirmed case are candidates for antimicrobial chemoprophylaxis regardless of vaccination status [25]; failure to obtain an exposure history therefore represents a missed opportunity to identify individuals requiring urgent assessment and post-exposure management. Similarly, referral was observed in only 30.5% of visits despite being reported by 78.4% of participants, while symptomatic medication was dispensed in 88.9% of encounters. These findings highlight the need to strengthen structured risk assessment and timely referral, while recognizing that symptomatic medication provision and referral were not mutually exclusive outcomes. These findings are broadly consistent with previous simulated patient research in Saudi community pharmacies reporting limitations in pharmacists’ counselling practices [8]. Importantly, symptomatic treatment is not inherently inappropriate and may be clinically justified in many situations; the concern is not the provision of symptomatic relief itself, but its apparent predominance over exposure assessment, red-flag recognition, and referral when a potentially serious infectious syndrome is present.

The simulated patient scenario evaluated recognition and management of a symptomatic presentation, including exposure-related enquiry, urgent referral, and preventive advice. It did not directly assess post-exposure antimicrobial prophylaxis for asymptomatic close contacts.

### 4.4. Associations Between Knowledge, Attitude, and Practice Domains

The correlation analyses provide further insight into the interrelationships among the three domains. Knowledge, attitudes, and self-reported practice scores were positively interrelated, with the strongest correlation between attitudes and practice (rho = 0.54) and weaker correlations involving knowledge (rho = 0.32–0.36). These associations are compatible with the general expectation, embedded in the knowledge–attitude–practice model, that knowledge and professional attitudes are related to reported practice. However, the sociodemographic and professional factors examined explained only 7.5%, 6.3%, and 4.6% of the variance in knowledge, attitudes, and practices scores, respectively, leaving a substantial proportion of variation unexplained and implying that these constructs are multidimensional and shaped by determinants not captured here, such as prior infectious-disease training, access to clinical protocols, organizational support, and individual clinical experience. These findings should not be read as evidence that knowledge or attitudes causally improve practice: because the design was cross-sectional, temporal precedence cannot be established and reciprocal relationships are plausible, since repeated practice may reinforce knowledge and attitudes just as favorable attitudes may facilitate engagement with training. The observed associations between a doctoral qualification and lower knowledge, and between female sex and lower attitude scores, and between 1–5 years of experience and lower attitude and practice scores, warrant equal caution; rather than reflecting intrinsic differences, these associations may arise from subgroup size, differing professional roles, or unmeasured confounders, and would require replication in larger and more balanced samples before being considered robust.

### 4.5. Regional Variation in Observed Behavior

Regional differences were evident across several simulated patient outcomes, with Taif consistently demonstrating the lowest observed performance: pharmacists in Taif showed no recognition of possible meningococcal disease, no enquiry about close contact, and no documented referral, while symptomatic medication was provided in every Taif encounter. These contrasts may reflect contextual differences across practice environments; however, the underlying reasons cannot be established from the present data. Possible explanations could include variation in training, patient volume, workload, access to referral pathways, or local organizational policies. Importantly, these factors were not directly measured and should therefore be regarded as hypotheses for future investigation rather than established explanations for the observed regional differences. A comparable pattern of geographical heterogeneity was observed in a previous study of drug–drug interaction management conducted in the same region, in which both the frequency of exposure and the rate of hazard detection differed significantly by city, and in which Makkah’s distinctive Hajj-related caseload was reflected in higher daily exposure [13]; the well-recognized intensity of clinical demand in the holy cities during mass gatherings has likewise been described in clinical pharmacy task-force reports [26]. These regional findings should nonetheless be regarded as exploratory and hypothesis-generating rather than as evidence that pharmacists in any one city are inherently more or less competent, particularly because the number of simulated visits differed between regions and some percentages may consequently be unstable.

### 4.6. Implications for Pharmacy Practice and Public Health

The juxtaposition of favorable knowledge and attitudes with poor observed performance carries a clear educational implication: strategies that focus primarily on the transmission of factual knowledge are unlikely to be sufficient when the principal limitation lies in translating knowledge into action during a real encounter. Pharmacy education and continuing professional development should therefore incorporate experiential and simulation-based approaches that develop clinical reasoning, communication, and practical decision-making skills [27], incorporating case-based learning, red-flag symptom recognition, structured exposure-history taking, structured counselling and referral algorithms, and simulation-based training targeted at pharmacists involved in Hajj- and Umrah-related care. This orientation is supported by systematic-review evidence indicating that multifaceted interventions, combining educational meetings, materials, outreach, feedback, and practice guidelines, can improve pharmacist counselling behavior, although the certainty of the evidence varies across studies [28].

Beyond education, system-level support is likely to be necessary to close the observed gap. A standardized pharmacy-based counselling and referral pathway, providing a brief checklist that prompts assessment of red-flag symptoms, recent close contact, the need for urgent referral, and appropriate preventive advice, could offer structured decision support at the point of care and would be especially valuable during mass gatherings, when pharmacists frequently serve as the first point of contact for pilgrims. This would represent a proposed intervention rather than currently established standardized pathway across Saudi community pharmacies. Where digital infrastructure permits, clinical decision-support tools may reinforce such pathways, provided that alerts are designed with attention to specificity and workflow integration to avoid alert fatigue [21]. Effective escalation also depends on robust interprofessional communication, since limitations in pharmacist–prescriber communication are a recognized obstacle to timely referral [29,30], and on adequate staffing, because workload pressures and workforce-retention challenges constrain the time available for structured assessment [31]. Although organizational strategies addressing workload and staffing may be relevant, the present study did not establish workload or time pressure as causes of the observed performance gap. The feasibility and value of embedding enhanced clinical-pharmacy services within the mass gathering healthcare response have been demonstrated during recent Hajj seasons [26], suggesting that the interventions proposed here are realistic within the Saudi context.

### 4.7. Strengths and Limitations

This study has several strengths. First, it addressed a specific and clinically relevant gap by examining pharmacists’ knowledge, attitudes, and practices related to meningococcal disease management during mass gatherings rather than general infectious-disease knowledge. Second, it combined a self-administered knowledge–attitude–practice survey with covert simulated patient encounters, allowing both reported and observed dimensions of practice to be examined and reducing reliance on self-reported data alone. Third, the inclusion of pharmacists from four cities in Western Saudi Arabia provided geographical diversity within the study setting. Fourth, multivariable modelling was used to identify factors associated with knowledge, attitudes, and self-reported practice while accounting for several demographic and professional characteristics, with robust standard errors and sensitivity analyses used to assess the stability of the estimates.

Several limitations should also be acknowledged. First, the cross-sectional design precludes causal inference, and the regression findings should be interpreted as associations rather than causal effects. Second, the survey component relied on self-reported responses and may have been affected by recall and social-desirability biases. Furthermore, because the survey was open-book, knowledge scores may partly reflect access to reference materials rather than unaided recall. In addition, the internal consistency of the knowledge domain was modest (Cronbach’s alpha = 0.631), reflecting the small number of heterogeneous binary items; the total knowledge score should therefore be interpreted together with the item-level results.

Third, because individual survey responses could not be linked to simulated patient encounters, the observed discrepancy represents a group-level rather than an individual-level comparison and should not be interpreted as demonstrating that the same pharmacists who reported favorable practices subsequently failed to perform them. Additionally, the survey included both community and hospital pharmacists, whereas simulated patient encounters were conducted exclusively in community pharmacies. Differences between practice settings may therefore have influenced the observed discrepancy.

Fourth, the use of a single standardized simulated patient scenario may not capture the full range of clinical presentations encountered in routine practice, limiting the generalizability of the observed findings. Formal inter-rater reliability was not assessed, as each simulated patient also served as the assessor. Although the visits were covert and unannounced to minimize the Hawthorne effect, its occurrence cannot be entirely excluded.

Fifth, regional comparisons involved multiple statistical tests (although Holm-adjusted *p*-values are reported) and unequal numbers of visits across cities, increasing the possibility of unstable estimates or chance findings. Furthermore, because each simulated patient was assigned to a single city, simulated patient effects cannot be fully separated from city effects. Regional comparisons should therefore be interpreted as exploratory.

Sixth, although the study included pharmacists from four cities in Western Saudi Arabia, the findings may have limited generalizability to other regions of Saudi Arabia. In addition, the observed practice findings cannot be directly generalized to hospital pharmacy settings because simulated patient encounters were conducted only in community pharmacies.

Finally, several potentially relevant contextual factors, including previous meningococcal training, workload, availability of referral protocols, and organizational support, were not directly measured and could not be adjusted for.

### 4.8. Future Research

Future research should determine whether targeted educational and practice-based interventions can improve observed behavior rather than merely raising knowledge or self-reported practice scores, ideally through repeated simulated patient assessments conducted before and after an intervention. Where feasible, future studies should link survey responses to individual simulated patient encounters, enabling direct measurement of concordance between reported and observed practice. Additional work should investigate the contextual determinants of behavior, including workload, prior training, access to clinical decision-support tools, availability of standardized referral pathways, and previous experience with suspected infectious disease. Finally, larger multicenter studies spanning a greater number of pharmacies across Saudi Arabia would help establish whether the regional differences observed here are reproducible.

## 5. Conclusions

In conclusion, the present study identified a substantial group-level discrepancy between favorable self-reported practices and observed performance during simulated patient encounters involving suspected meningococcal disease. Although pharmacists generally demonstrated relatively high knowledge and favorable attitudes toward their professional responsibilities, these strengths were not consistently translated into disease recognition, exposure assessment, referral, or post-exposure preventive counselling in practice. The findings highlight the importance of evaluating pharmacy practice using both self-reported and observed measures, particularly for high-risk conditions requiring rapid recognition and referral. Strengthening practical competency through targeted training, standardized counselling and referral approaches, and system-level support may help improve pharmacists’ readiness to contribute effectively to meningococcal disease prevention during mass gatherings.

## Figures and Tables

**Table 1:** Demographic and professional characteristics.

Characteristic	Description, N = 380—*n* (%)
Gender	
Male	212 (55.8%)
Female	168 (44.2%)
Age	
23 to 28	250 (65.8%)
29 to 35	93 (24.5%)
36 to 40	29 (7.6%)
41 and above	8 (2.1%)
Highest pharmacy-related educational qualification	
Bachelor (B.Pharm)	111 (29.2%)
Bachelor (Pharm.D)	216 (56.8%)
Master	38 (10.0%)
Doctor of Philosophy (PhD)	15 (3.9%)
Current practice setting	
Community pharmacy	206 (54.2%)
Hospital pharmacy	174 (45.8%)
City of current practice	
Makkah	101 (26.6%)
Jeddah	117 (30.8%)
Taif	86 (22.6%)
Madinah	76 (20.0%)
Years of experience as a licensed pharmacist	
Less than 1 year	136 (35.8%)
1–5 years	163 (42.9%)
6–10 years	41 (10.8%)
More than 10 years	40 (10.5%)

**Table 2:** Participants’ Responses Regarding Knowledge about Meningococcal Disease and Post-Exposure Prevention.

Characteristic	Description, N = 380—*n* (%)
Organism which causes the meningococcal disease	
*Neisseria meningitidis* *	304 (80.0%)
Haemophilus influenzae	10 (2.6%)
*Streptococcus pneumoniae*	45 (11.8%)
I do not know	21 (5.5%)
Close contact with an infected person increases the risk of meningococcal transmission	
True *	313 (82.4%)
False	49 (12.9%)
I do not know	18 (4.7%)
Mandatory meningococcal vaccination completely eliminates the risk of disease transmission	
True	161 (42.4%)
False *	186 (48.9%)
I do not know	33 (8.7%)
Post-exposure preventive counselling remains necessary even among vaccinated individuals	
True *	315 (82.9%)
False	28 (7.4%)
I do not know	37 (9.7%)
Which of the following symptom clusters is most suggestive of meningococcal disease and requires immediate medical referral?	
High fever, severe headache, neck stiffness, and a purpuric rash *	311 (81.8%)
Abdominal pain, nausea, vomiting, and diarrhea	14 (3.7%)
Low-grade fever, runny nose, sore throat, and cough	28 (7.4%)
Fatigue, mild headache, muscle pain, and dizziness	7 (1.8%)
I do not know	20 (5.3%)
What is the recommended action for a patient recently exposed to a confirmed case of meningococcal disease?	
Immediate referral to a healthcare facility *	256 (67.4%)
Provide prophylactic antibiotics without referral	74 (19.5%)
Advise monitoring at home only	34 (8.9%)
I do not know	16 (4.2%)

* Correct response. Percentages may not sum to exactly 100% because of rounding.

**Table 3:** Descriptive statistics of the domains under study.

Characteristic	Median (Q1–Q3)	Mean ± SD	Min–Max	Cronbach’s Alpha (95% CI)	N of Items	McDonald’s Omega
Knowledge	5.0 (4.0–6.0)	4.4 ± 1.5	0.0–6.0	0.631 (0.570–0.685)	6	0.673
Attitudes	13.0 (10.0–14.0)	12.1 ± 2.6	0.0–15.0	0.756 (0.715–0.793)	5	0.758
Practices	15.5 (13.0–18.0)	15.0 ± 4.2	0.0–20.0	0.809 (0.777–0.838)	5	0.812

**Table 4:** Factors associated with knowledge scores (N = 380).

Characteristic	Univariable Regression			Multivariable Regression *		
	Beta	95% CI	*p*-Value	Beta	95% CI	*p*-Value
Gender						
Male	Reference	Reference		Reference	Reference	
Female	−0.24	−0.55, 0.06	0.119	−0.26	−0.59, 0.07	0.122
Age						
23 to 28	Reference	Reference		Reference	Reference	
29 to 35	−0.07	−0.44, 0.29	0.693	0.14	−0.33, 0.60	0.561
36 to 40	−0.23	−0.84, 0.38	0.460	0.32	−0.63, 1.27	0.509
41 and above	−0.10	−1.23, 1.04	0.867	0.25	−1.20, 1.70	0.733
Highest pharmacy-related educational qualification						
Bachelor (B.Pharm)	Reference	Reference		Reference	Reference	
Bachelor (Pharm.D)	0.04	−0.30, 0.39	0.811	−0.05	−0.43, 0.33	0.799
Master	−0.35	−0.92, 0.22	0.232	−0.39	−0.97, 0.20	0.196
Doctor of Philosophy (PhD)	−1.50	−2.59, −0.41	0.007	−1.55	−2.76, −0.34	0.012
Current practice setting						
Community pharmacy	Reference	Reference		Reference	Reference	
Hospital pharmacy	0.10	−0.20, 0.40	0.516	0.27	−0.05, 0.59	0.100
City of current practice						
Makkah	Reference	Reference		Reference	Reference	
Jeddah	−0.04	−0.46, 0.38	0.852	−0.03	−0.47, 0.41	0.890
Taif	0.04	−0.38, 0.46	0.855	0.17	−0.27, 0.61	0.445
Madinah	0.41	0.00, 0.82	0.052	0.44	0.03, 0.84	0.035
Years of experience as a licensed pharmacist						
Less than 1 year	Reference	Reference		Reference	Reference	
1–5 years	−0.24	−0.57, 0.10	0.164	−0.33	−0.69, 0.02	0.068
6–10 years	−0.31	−0.88, 0.26	0.286	−0.43	−1.14, 0.28	0.234
More than 10 years	−0.53	−1.06, 0.01	0.054	−0.46	−1.41, 0.49	0.341

All 380 participants were analysed. The multivariable model included all six factors and explained 0.075 (R^2^) or 0.039 (adjusted R^2^) of the variance. Model diagnostics: independence of observations by design (one response per pharmacist); Breusch–Pagan *p* = 0.237; Shapiro–Wilk test for residuals *p* < 0.001; maximum GVIF^1/(2×Df)^ = 1.21; 26 observations had Cook’s distance > 4/*n*. 95% CIs and *p*-values are based on HC3 heteroscedasticity-consistent robust standard errors. * All six factors were entered simultaneously in the multivariable model. Sensitivity analyses (bootstrap confidence intervals and exclusion of influential observations) are presented in Table S2. Abbreviation: CI = Confidence Interval.

**Table 5:** Factors associated with attitude scores (N = 380).

Characteristic	Univariable Regression			Multivariable Regression *		
	Beta	95% CI	*p*-Value	Beta	95% CI	*p*-Value
Gender						
Male	Reference	Reference		Reference	Reference	
Female	−0.96	−1.50, −0.43	<0.001	−0.94	−1.52, −0.35	0.002
Age						
23 to 28	Reference	Reference		Reference	Reference	
29 to 35	0.01	−0.59, 0.61	0.978	0.28	−0.49, 1.05	0.482
36 to 40	0.32	−0.71, 1.36	0.540	0.44	−1.20, 2.08	0.597
41 and above	0.69	−1.46, 2.85	0.527	0.25	−2.45, 2.94	0.858
Highest pharmacy-related educational qualification						
Bachelor (B.Pharm)	Reference	Reference		Reference	Reference	
Bachelor (Pharm.D)	0.38	−0.22, 0.97	0.212	0.39	−0.25, 1.03	0.237
Master	−0.21	−1.28, 0.86	0.702	−0.07	−1.14, 1.00	0.897
Doctor of Philosophy (PhD)	−0.39	−2.01, 1.24	0.641	−0.56	−2.33, 1.20	0.532
Current practice setting						
Community pharmacy	Reference	Reference		Reference	Reference	
Hospital pharmacy	−0.11	−0.64, 0.43	0.701	0.01	−0.59, 0.61	0.974
City of current practice						
Makkah	Reference	Reference		Reference	Reference	
Jeddah	−0.18	−0.88, 0.52	0.613	−0.04	−0.77, 0.69	0.908
Taif	−0.21	−0.95, 0.52	0.574	0.13	−0.66, 0.91	0.753
Madinah	0.36	−0.43, 1.15	0.373	0.55	−0.24, 1.34	0.171
Years of experience as a licensed pharmacist						
Less than 1 year	Reference	Reference		Reference	Reference	
1–5 years	−0.55	−1.13, 0.03	0.063	−0.70	−1.35, −0.05	0.035
6–10 years	−0.47	−1.48, 0.55	0.369	−0.78	−1.99, 0.43	0.208
More than 10 years	0.16	−0.77, 1.09	0.740	0.05	−1.45, 1.55	0.944

All 380 participants were analysed. The multivariable model included all six factors and explained 0.063 (R^2^) or 0.027 (adjusted R^2^) of the variance. Model diagnostics: independence of observations by design (one response per pharmacist); Breusch–Pagan *p* = 0.901; Shapiro–Wilk test for residuals *p* < 0.001; maximum GVIF^1/(2×Df)^ = 1.21; 20 observations had Cook’s distance > 4/*n*. 95% CIs and *p*-values are based on HC3 heteroscedasticity-consistent robust standard errors. * All six factors were entered simultaneously in the multivariable model. Sensitivity analyses (bootstrap confidence intervals and exclusion of influential observations) are presented in Table S2. Abbreviation: CI = Confidence Interval.

**Table 6:** Factors associated with practice scores (N = 380).

Characteristic	Univariable Regression			Multivariable Regression *		
	Beta	95% CI	*p*-Value	Beta	95% CI	*p*-Value
Gender						
Male	Reference	Reference		Reference	Reference	
Female	−0.73	−1.61, 0.15	0.102	−0.97	−1.96, 0.01	0.053
Age						
23 to 28	Reference	Reference		Reference	Reference	
29 to 35	−0.44	−1.48, 0.60	0.407	0.20	−1.05, 1.45	0.754
36 to 40	1.05	−0.47, 2.56	0.175	1.60	−0.76, 3.96	0.184
41 and above	0.89	−1.11, 2.88	0.383	1.16	−2.12, 4.45	0.487
Highest pharmacy-related educational qualification						
Bachelor (B.Pharm)	Reference	Reference		Reference	Reference	
Bachelor (Pharm.D)	−0.07	−1.05, 0.91	0.891	−0.21	−1.33, 0.92	0.716
Master	0.02	−1.46, 1.49	0.982	0.32	−1.32, 1.96	0.704
Doctor of Philosophy (PhD)	−0.50	−2.47, 1.47	0.617	−0.85	−3.00, 1.30	0.439
Current practice setting						
Community pharmacy	Reference	Reference		Reference	Reference	
Hospital pharmacy	−0.16	−1.03, 0.70	0.712	0.05	−0.88, 0.99	0.913
City of current practice						
Makkah	Reference	Reference		Reference	Reference	
Jeddah	−0.29	−1.53, 0.94	0.641	−0.17	−1.47, 1.14	0.803
Taif	0.38	−0.84, 1.59	0.542	0.58	−0.69, 1.84	0.370
Madinah	0.12	−1.20, 1.44	0.860	0.37	−0.98, 1.73	0.588
Years of experience as a licensed pharmacist						
Less than 1 year	Reference	Reference		Reference	Reference	
1–5 years	−1.35	−2.30, −0.40	0.005	−1.54	−2.61, −0.47	0.005
6–10 years	−1.21	−2.53, 0.11	0.072	−1.86	−3.51, −0.20	0.028
More than 10 years	−0.30	−1.81, 1.22	0.702	−1.41	−4.08, 1.26	0.300

All 380 participants were analysed. The multivariable model included all six factors and explained 0.046 (R^2^) or 0.010 (adjusted R^2^) of the variance. Model diagnostics: independence of observations by design (one response per pharmacist); Breusch–Pagan *p* = 0.075; Shapiro–Wilk test for residuals *p* < 0.001; maximum GVIF^1/(2×Df)^ = 1.21; 19 observations had Cook’s distance > 4/*n*. 95% CIs and *p*-values are based on HC3 heteroscedasticity-consistent robust standard errors. * All six factors were entered simultaneously in the multivariable model. Sensitivity analyses (bootstrap confidence intervals and exclusion of influential observations) are presented in Table S2. Abbreviation: CI = Confidence Interval.

**Table 7:** Participants’ responses to simulated patient records based on the overall statistics and regional differences.

Characteristic	Overall N = 305	Makkah N = 100	Madinah N = 60	Jeddah N = 85	Taif N = 60	*p*-Value
The pharmacist recognized the possibility of meningococcal disease based on the presented symptoms	16 (5.2%) [3.0–8.4]	5 (5.0%) [1.6–11.3]	2 (3.3%) [0.4–11.5]	9 (10.6%) [5.0–19.2]	0 (0.0%) [0.0–6.0]	0.034 ^a^ (0.101)
The pharmacist suspected an infection without specifying meningococcal disease	92 (30.2%) [25.1–35.7]	20 (20.0%) [12.7–29.2]	30 (50.0%) [36.8–63.2]	42 (49.4%) [38.4–60.5]	0 (0.0%) [0.0–6.0]	<0.001 ^b^ (<0.001)
The pharmacist asked about recent close contact with a suspected or confirmed meningococcal case after the SP described suggestive symptoms	23 (7.5%) [4.8–11.1]	8 (8.0%) [3.5–15.2]	3 (5.0%) [1.0–13.9]	12 (14.1%) [7.5–23.4]	0 (0.0%) [0.0–6.0]	0.007 ^a^ (0.036)
The pharmacist advised immediate referral to a healthcare facility	93 (30.5%) [25.4–36.0]	15 (15.0%) [8.6–23.5]	35 (58.3%) [44.9–70.9]	43 (50.6%) [39.5–61.6]	0 (0.0%) [0.0–6.0]	<0.001 ^a^ (<0.001)
The pharmacist provided advice on preventing further transmission following suspected exposure	19 (6.2%) [3.8–9.6]	8 (8.0%) [3.5–15.2]	5 (8.3%) [2.8–18.4]	3 (3.5%) [0.7–10.0]	3 (5.0%) [1.0–13.9]	0.514 ^a^ (0.514)
The pharmacist provided medications for symptomatic relief	271 (88.9%) [84.8–92.2]	85 (85.0%) [76.5–91.4]	52 (86.7%) [75.4–94.1]	74 (87.1%) [78.0–93.4]	60 (100.0%) [94.0–100.0]	0.022 ^b^ (0.090)
The pharmacist’s management (if inappropriate) was primarily affected by time pressure or high patient volume	19 (6.2%) [3.8–9.6]	9 (9.0%) [4.2–16.4]	3 (5.0%) [1.0–13.9]	7 (8.2%) [3.4–16.2]	0 (0.0%) [0.0–6.0]	0.063 ^a^ (0.126)

*n* (%) [exact Clopper–Pearson 95% CI]. ^a^ denotes using Fisher’s exact test; ^b^ indicates using Pearson’s Chi-squared test. Holm-adjusted *p*-values for the seven regional comparisons are shown in parentheses; regional comparisons are exploratory.

## Data Availability

The data presented in this study are available from the corresponding author upon reasonable request. The data are not publicly available due to privacy and ethical considerations.

## Supplementary Materials

The following supporting information can be downloaded at https://www.mdpi.com/article/10.3390/healthcare14213765/s1: Questionnaire S1: Survey questionnaire assessing pharmacists’ knowledge, attitudes, and practices regarding meningococcal disease recognition, referral, and post-exposure preventive counselling during mass gatherings; Supplementary Materials S2: Standardized Simulated-Patient Script and Post-Visit Checklist; STROBE Checklist S3: STROBE checklist; Table S1: Reliability and item analysis of the knowledge, attitude, and practice domains; Table S2: Sensitivity analyses of the multivariable linear regression models.
