# Supplementary material for: The Knowledge–Practice Gap in Pharmacists’ Management of Suspected Meningococcal Disease During Mass Gatherings: Evidence from Surveys and Simulated Patient Visits
Source: Healthcare (Basel). 2026 Nov 4;14(21):3765. doi: 10.3390/healthcare14213765 (PMC13762988; doi:10.3390/healthcare14213765)
Supplement: Supplementary file 1 [file healthcare-14-03765-s001.zip › Questionnaire_S1.pdf]

Pharmacists’ Role in Meningococcal Disease Prevention at Mass Gatherings

- This study aims to
  assess pharmacists’ knowledge, attitudes, and practices in post-exposure preventive counselling
  for meningococcal disease during mass gatherings in Saudi Arabia.

- Participation is voluntary, takes about 3-4 minutes, and all responses are anonymous and confidential. By continuing, you provide your consent to participate.

* Indicates required question

Section 1: Demographics

1. **1. Gender:** *

Mark only one oval.

- Male
- Female

2. **2. Age:** *

Mark only one oval.

- 23 - 28
- 29 - 35
- 36 - 40
- 41 and above

3. **3. Highest pharmacy-related educational qualification:** *

Mark only one oval.

- Bachelor (B.Pharm)
- Bachelor (Pharm.D)
- Master
- Doctor of philosophy (PhD)

4. **4. Current practice setting:** *

Mark only one oval.

- Community pharmacy
- Hospital pharmacy

5. **5. City of current practice:** *

Mark only one oval.

- Makkah
- Jeddah
- Madinah
- Taif

6. **6. Years of experience as a licensed pharmacist:** *

Mark only one oval.

- Less than 1 year
- 1-5 years
- 6-10 years
- More than 10 years

Section 2: Knowledge about Meningococcal Disease and Post-Exposure Prevention

7. **1. Which organism causes meningococcal disease?** *

Mark only one oval.

- Neisseria meningitidis
- Streptococcus pneumoniae
- Haemophilus influenzae
- I do not know

8. **2. Close contact with an infected person increases the risk of meningococcal transmission.** *

Mark only one oval.

- True
- False
- I do not know

9. **3. Mandatory meningococcal vaccination completely eliminates the risk of disease transmission.** *

Mark only one oval.

- True
- False
- I do not know

10. **4. Post-exposure preventive counselling remains necessary even among vaccinated individuals.** *

Mark only one oval.

- True
- False
- I do not know

11. **5. Which of the following symptom clusters is most suggestive of meningococcal disease and requires immediate medical referral?** *

Mark only one oval.

- High fever, severe headache, neck stiffness, and a purpuric rash
- Low-grade fever, runny nose, sore throat, and cough
- Abdominal pain, nausea, vomiting, and diarrhea
- Fatigue, mild headache, muscle pain, and dizziness
- I do not know

12. **6. What is the recommended action for a patient recently exposed to a confirmed case of meningococcal disease?** *

Mark only one oval.

- Advise monitoring at home only
- Immediate referral to a healthcare facility
- Provide prophylactic antibiotics without referral
- I do not know

Section 3: Attitudes toward Post-Exposure Preventive Counselling for Meningococcal Disease

13. **1. Pharmacists have an important role in preventing meningococcal disease during mass gatherings.** *

Mark only one oval.

- Strongly agree
- Agree
- Disagree
- Strongly disagree

14. **2. Providing post-exposure preventive counselling is part of my professional responsibility as a pharmacist.** *

Mark only one oval.

- Strongly agree
- Agree
- Disagree
- Strongly disagree

15. **3. Pharmacists should actively refer individuals with suspected meningococcal exposure for medical care.** *

Mark only one oval.

- Strongly agree
- Agree
- Disagree
- Strongly disagree

16. **4. Post-exposure preventive counselling can help reduce meningococcal disease transmission during Hajj and Umrah.** *

Mark only one oval.

- Strongly agree
- Agree
- Disagree
- Strongly disagree

17. **5. I feel confident in providing post-exposure preventive counselling for meningococcal disease.** *

Mark only one oval.

- Strongly agree
- Agree
- Disagree
- Strongly disagree

Section 4: Post-Exposure Preventive Counselling Practices during Mass Gatherings

Please answer based on your routine practice during the **past 3 months** in your current workplace.

18. **1. I ask patients about recent close contact with a confirmed or suspected meningococcal case when they present with symptoms suggestive of meningococcal disease.** *

Mark only one oval.

- Always (>75%)
- Often (51–75%)
- Sometimes (26–50%)
- Rarely (≤25%)
- Never (0%)

19. **2. When a patient presents with clinical features suggestive of meningococcal disease (e.g., fever, neck stiffness, petechial rash), I consider meningococcal infection in my clinical assessment.** *

Mark only one oval.

- Always (>75%)
- Often (51–75%)
- Sometimes (26–50%)
- Rarely (≤25%)
- Never (0%)

20. **3. I advise immediate medical referral when meningococcal disease is suspected.** *

Mark only one oval.

- Always (>75%)
- Often (51–75%)
- Sometimes (26–50%)
- Rarely (≤25%)
- Never (0%)

21. **4. I provide counselling on infection prevention measures (e.g., hygiene practices, minimizing close contact, and awareness of warning symptoms) following suspected meningococcal exposure.** *

Mark only one oval.

- Always (>75%)
- Often (51–75%)
- Sometimes (26–50%)
- Rarely (≤25%)
- Never (0%)

22. **5. During mass gatherings, I provide post-exposure preventive counselling despite time constraints and workload.** *

Mark only one oval.

- Always (>75%)
- Often (51–75%)
- Sometimes (26–50%)
- Rarely (≤25%)
- Never (0%)
