# Supplementary material for: The Knowledge–Practice Gap in Pharmacists’ Management of Suspected Meningococcal Disease During Mass Gatherings: Evidence from Surveys and Simulated Patient Visits
Source: Healthcare (Basel). 2026 Nov 4;14(21):3765. doi: 10.3390/healthcare14213765 (PMC13762988; doi:10.3390/healthcare14213765)
Supplement: Supplementary file 1 [file healthcare-14-03765-s001.zip › STROBE_Checklist_S3.pdf]

STROBE Statement — Checklist of items that should be included in reports of cross-sectional studies

The Knowledge–Practice Gap in Pharmacists' Management of Suspected Meningococcal Disease During Mass Gatherings: Evidence from Surveys and Simulated Patient Visits

Section/Topic	Item	Recommendation	Reported in (section)	Page
Title and abstract	1	(a) Indicate the study's design with a commonly used term in the title or the abstract	Abstract	1
		(b) Provide in the abstract an informative and balanced summary of what was done and what was found	Abstract	1
**Introduction**				
Background/rationale	2	Explain the scientific background and rationale for the investigation being reported	1. Introduction	2–3
Objectives	3	State specific objectives, including any prespecified hypotheses	1. Introduction — final paragraph	3
**Methods**				
Study design	4	Present key elements of study design early in the paper	2.1. Study Design	4
Setting	5	Describe the setting, locations, and relevant dates, including periods of recruitment, exposure, follow-up, and data collection	2.2. Study Setting and Participants; 2.4. Simulated Patient (SP) Visits	4–6
Participants	6	Give the eligibility criteria, and the sources and methods of selection of participants	2.2. Study Setting and Participants; 2.4. SP Visits	4, 6
Variables	7	Clearly define all outcomes, exposures, predictors, potential confounders, and effect modifiers	2.3. Survey Instrument; 2.4. SP Visits; 2.6. Scoring; 2.7. Statistical Analysis	5–8
Data sources/measurement	8	For each variable of interest, give sources of data and details of methods of assessment (measurement)	2.3. Survey Instrument	5–7
Bias	9	Describe any efforts to address potential sources of bias	2.3. Survey Instrument — expert review and pilot testing; 2.4. SP Visits — standardization and covert encounters; 4.7. Strengths and Limitations — potential biases	5–7, 26–28
Study size	10	Explain how the study size was arrived at	2.5. Sample Size Calculation	7
Quantitative variables	11	Explain how quantitative variables were handled in the analyses. If applicable, describe which groupings were chosen, and why	2.3. Survey Instrument; 2.6. Scoring; 2.7. Statistical Analysis; Table 1 — coding, score ranges, and categories	5, 7–9
Statistical methods	12	(a) Describe all statistical methods, including those used to control for confounding	2.7. Statistical Analysis	7–8
		(b) Describe any methods used to examine subgroups and interactions	2.7. Statistical Analysis — exploratory city-level comparisons; interaction analyses not reported	8
		(c) Explain how missing data were addressed	2.7. Statistical Analysis	8
		(d) If applicable, describe analytical methods taking account of sampling strategy	2.2. Study Setting and Participants; 2.4. SP Visits	4, 6
		(e) Describe any sensitivity analyses	2.7. Statistical Analysis	8, 14–18

Section/Topic	Item	Recommendation	Reported in (section)	Page
**Results**				
Participants	13	(a) Report numbers of individuals at each stage of the study	2.2. Study Setting and Participants; 2.4. SP Visits; 3.1. Demographic and Professional Characteristics; 3.9. SP Observations	4–6, 9, 18–19
		(b) Give reasons for non-participation at each stage	2.4. SP Visits — no refusals or incomplete encounters; Survey — reasons for non-participation were not recoverable from the survey platform.	6
		(c) Consider use of a flow diagram	No participant flow diagram; survey and SP sample totals are described in Sections 2.4, 3.1, and 3.9	6, 9, 18
Descriptive data	14	(a) Give characteristics of study participants and information on exposures and potential confounders	3.1. Demographic and Professional Characteristics; Table 1; 3.9. SP Observations and Table 7	9, 18–19
		(b) Indicate number of participants with missing data for each variable of interest	2.7. Statistical Analysis — no missing values in the final analytic dataset	8
Outcome data	15	Report numbers of outcome events or summary measures	3.2–3.5 — survey knowledge, attitudes, practices, and domain summaries; 3.9. SP Observations; Tables 2, 3, and 7	10–14, 18–19
Main results	16	(a) Give unadjusted and adjusted estimates and their precision (e.g., 95% CI). Make clear which confounders were adjusted for and why	2.7. Statistical Analysis; 3.6–3.8 — univariable and multivariable regression with 95% CIs; Tables 4–6	7–8, 14–18
		(b) Report category boundaries when continuous variables were categorized	2.3. Survey Instrument; 2.6. Scoring — response categories and score ranges; Table 1; Tables 4–6	5, 7, 9, 15–17
		(c) If relevant, consider translating estimates of relative risk into absolute risk	Not applicable	—
Other analyses	17	Report other analyses done — e.g., analyses of subgroups and interactions and sensitivity analyses	2.7. Statistical Analysis; 3.6–3.9 — sensitivity and exploratory regional analyses; Table 7 and Table S2	8, 14–19
**Discussion**				
Key results	18	Summarise key results with reference to study objectives	4. Discussion — opening paragraph	20
Limitations	19	Discuss limitations of the study, taking into account sources of potential bias or imprecision	4.7. Strengths and Limitations	26–28
Interpretation	20	Give a cautious overall interpretation of results considering objectives, limitations, multiplicity of analyses, and other relevant evidence	4. Discussion (Sections 4.1–4.8); 5. Conclusions	20–28
Generalisability	21	Discuss the generalisability (external validity) of the study results	4.7. Strengths and Limitations	27–28
**Other information**				
Funding	22	Give the source of funding and the role of the funders for the present study	Funding	29

An Explanation and Elaboration article discusses each checklist item and gives methodological background and published examples of transparent reporting. The STROBE checklist is best used in conjunction with that article, freely available on the websites of Annals of Internal Medicine, PLoS Medicine, and Epidemiology. Information on the STROBE Initiative is available at www.strobe-statement.org.
