# Supplementary material for: The Knowledge–Practice Gap in Pharmacists’ Management of Suspected Meningococcal Disease During Mass Gatherings: Evidence from Surveys and Simulated Patient Visits
Source: Healthcare (Basel). 2026 Nov 4;14(21):3765. doi: 10.3390/healthcare14213765 (PMC13762988; doi:10.3390/healthcare14213765)
Supplement: Supplementary file 1 [file healthcare-14-03765-s001.zip › Supplementary Material S2.pdf]

Supplementary Material S2

Standardized Simulated-Patient Script and Post-Visit Checklist

This supplement documents the symptom-based script and the structured checklist used in the simulated-patient (SP) visits. The script is presented in English translation from the Arabic-language encounter instructions.

A. Simulated-patient visit script

Scenario: A customer who recently returned from Umrah visits a community pharmacy seeking symptomatic relief. Symptoms are disclosed progressively; the SP does not independently suggest a diagnosis or connect the complaints.

SP — opening request:

> *"Since yesterday, I've had a fever and a really severe headache. I returned from Umrah two days ago. My neck hurts, and my whole body aches. I'd like something for the pain."*

SP — additional symptom: (if the pharmacist goes to retrieve or dispense medication)

> *"Sorry, I can't see very well; the light is too bright."*

SP — separate request: (subsequently)

> *"Oh, and I also need a cream or ointment for an allergy. I have small red spots on my back."*

At this stage, the SP showed the pharmacist a clinical image of a meningococcal disease–associated rash.

SP conduct and standardization

- Do not present all symptoms at once. Act as a patient who does not link the symptoms together.
- Do not suggest a diagnosis, provide medical hints, or imply that the rash is related to the headache or fever.
- Answer only what the pharmacist asks, in accordance with the standardized scenario practiced during training.
- Complete the checklist immediately after leaving the pharmacy.

B. Post-visit simulated-patient checklist

Completed by the SP immediately after leaving the pharmacy. Response options for each item: Yes / No.

1. Did the pharmacist recognize the possibility of meningococcal disease based on the presented symptoms?

☐ Yes ☐ No

2. Did the pharmacist suspect an infection without specifying meningococcal disease?

☐ Yes ☐ No

3. Did the pharmacist ask about recent close contact with a suspected or confirmed meningococcal case after the SP described suggestive symptoms?

☐ Yes ☐ No

4. Did the pharmacist advise immediate referral to a healthcare facility?

☐ Yes ☐ No

5. Did the pharmacist provide advice on preventing further transmission following suspected exposure?

☐ Yes ☐ No

6. Did the pharmacist provide medications for symptomatic relief?

☐ Yes ☐ No

7. Was the pharmacist's management (if inappropriate) primarily affected by time pressure or high patient volume?

☐ Yes ☐ No
