# Supplementary material for: The Knowledge–Practice Gap in Pharmacists’ Management of Suspected Meningococcal Disease During Mass Gatherings: Evidence from Surveys and Simulated Patient Visits
Source: Healthcare (Basel). 2026 Nov 4;14(21):3765. doi: 10.3390/healthcare14213765 (PMC13762988; doi:10.3390/healthcare14213765)
Supplement: Supplementary file 1 [file healthcare-14-03765-s001.zip › Supplementary Tables S1-S2 (statistics).pdf]

Table S1. Reliability and item analysis of the knowledge, attitude, and practice domains (N = 380).

Domain	Item	Mean (proportion correct for knowledge)	SD	Corrected item-total r	Alpha if item deleted
Knowledge	Organism which causes the meningococcal disease	0.80	0.40	0.44	0.557
Knowledge	Close contact with an infected person increases the risk of meningococcal transmission	0.82	0.38	0.44	0.561
Knowledge	Mandatory meningococcal vaccination completely eliminates the risk of disease transmission	0.49	0.50	0.24	0.647
Knowledge	Post-exposure preventive counselling remains necessary even among vaccinated individuals	0.83	0.38	0.42	0.568
Knowledge	Which of the following symptom clusters is most suggestive of meningococcal disease and requires immediate medical referral?	0.82	0.39	0.56	0.514
Knowledge	What is the recommended action for a patient recently exposed to a confirmed case of meningococcal disease?	0.67	0.47	0.17	0.666
**Knowledge**	**Domain: Cronbach's alpha = 0.631 (95% CI 0.570–0.685); McDonald's omega = 0.673**				
Attitudes	Pharmacists have an important role in preventing meningococcal disease during mass gatherings	2.43	0.68	0.52	0.715
Attitudes	Providing post-exposure preventive counselling is part of my professional responsibility as a pharmacist	2.39	0.75	0.54	0.707
Attitudes	Pharmacists should actively refer individuals with suspected meningococcal exposure for medical care	2.50	0.71	0.48	0.728
Attitudes	Post-exposure preventive counselling can help reduce meningococcal disease transmission during Hajj and Umrah	2.51	0.73	0.56	0.698
Attitudes	I feel confident in providing post-exposure preventive counselling for meningococcal disease	2.27	0.81	0.52	0.715
**Attitudes**	**Domain: Cronbach's alpha = 0.756 (95% CI 0.715–0.793); McDonald's omega = 0.758**				

Domain	Item	Mean (proportion correct for knowledge)	SD	Corrected item-total r	Alpha if item deleted
Practices	When a patient presents with clinical features suggestive of meningococcal disease, I state that the symptoms could indicate meningococcal disease.	2.89	1.07	0.48	0.805
Practices	I ask patients about recent close contact with a confirmed or suspected meningococcal case when they present with symptoms suggestive of meningococcal disease.	2.91	1.13	0.64	0.758
Practices	I advise immediate medical referral when meningococcal disease is suspected.	3.24	1.08	0.61	0.769
Practices	I provide counselling on infection prevention measures following suspected meningococcal exposure to prevent further transmission.	3.10	1.10	0.67	0.749
Practices	During mass gatherings, I provide post-exposure preventive counselling despite time constraints or high workload	2.84	1.22	0.59	0.776
**Practices**	**Domain: Cronbach's alpha = 0.809 (95% CI 0.777–0.838); McDonald's omega = 0.812**				

For knowledge items (scored 0/1), the mean is the proportion of correct responses. The alpha 95% CI was calculated using the Feldt method.

Table S2. Sensitivity analyses of the multivariable linear regression models (N = 380).

Outcome	Term	Main: β (95% CI, HC3)	Main p	Bootstrap 95% CI	Excl. influential: β (95% CI)	Excl. influential p
Knowledge	Gender: Female (ref: Male)	-0.26 (-0.59, 0.07)	0.122	-0.58, 0.06	-0.13 (-0.41, 0.16)	0.386
Knowledge	Age: 29 to 35 (ref: 23 to 28)	0.14 (-0.33, 0.60)	0.561	-0.31, 0.60	0.24 (-0.15, 0.63)	0.225
Knowledge	Age: 36 to 40 (ref: 23 to 28)	0.32 (-0.63, 1.27)	0.509	-0.53, 1.29	0.23 (-0.44, 0.90)	0.499
Knowledge	Age: 41 and above (ref: 23 to 28)	0.25 (-1.20, 1.70)	0.733	-1.15, 1.53	0.03 (-0.67, 0.72)	0.942
Knowledge	Highest pharmacy-related educational qualification: Bachelor (Pharm.D) (ref: Bachelor (B.Pharm))	-0.05 (-0.43, 0.33)	0.799	-0.40, 0.34	-0.09 (-0.41, 0.23)	0.573
Knowledge	Highest pharmacy-related educational qualification: Master (ref: Bachelor (B.Pharm))	-0.39 (-0.97, 0.20)	0.196	-0.99, 0.19	-0.45 (-0.96, 0.05)	0.080
Knowledge	Highest pharmacy-related educational qualification: Doctor of philosophy (PhD) (ref: Bachelor (B.Pharm))	-1.55 (-2.76, -0.34)	0.012	-2.62, -0.37	-2.23 (-3.18, -1.28)	<0.001
Knowledge	Current practice setting: Hospital pharmacy (ref: Community pharmacy)	0.27 (-0.05, 0.59)	0.100	-0.03, 0.59	0.18 (-0.10, 0.46)	0.208
Knowledge	City of current practice: Jeddah (ref: Makkah)	-0.03 (-0.47, 0.41)	0.890	-0.47, 0.39	0.12 (-0.24, 0.48)	0.517
Knowledge	City of current practice: Taif (ref: Makkah)	0.17 (-0.27, 0.61)	0.445	-0.26, 0.59	-0.03 (-0.44, 0.37)	0.868
Knowledge	City of current practice: Madinah (ref: Makkah)	0.44 (0.03, 0.84)	0.035	0.03, 0.81	0.36 (0.01, 0.72)	0.046
Knowledge	Years of experience as a licensed pharmacist: 1-5 years (ref: Less than 1 year)	-0.33 (-0.69, 0.02)	0.068	-0.67, 0.03	-0.33 (-0.63, -0.03)	0.034
Knowledge	Years of experience as a licensed pharmacist: 6-10 years (ref: Less than 1 year)	-0.43 (-1.14, 0.28)	0.234	-1.10, 0.27	-0.26 (-0.81, 0.28)	0.344
Knowledge	Years of experience as a licensed pharmacist: More than 10 years (ref: Less than 1 year)	-0.46 (-1.41, 0.49)	0.341	-1.42, 0.45	-0.07 (-0.64, 0.50)	0.808
Attitudes	Gender: Female (ref: Male)	-0.94 (-1.52, -0.35)	0.002	-1.53, -0.38	-1.00 (-1.53, -0.47)	<0.001
Attitudes	Age: 29 to 35 (ref: 23 to 28)	0.28 (-0.49, 1.05)	0.482	-0.50, 0.99	0.02 (-0.67, 0.71)	0.954

Outcome	Term	Main: β (95% CI, HC3)	Main p	Bootstrap 95% CI	Excl. influential: β (95% CI)	Excl. influential p
Attitudes	Age: 36 to 40 (ref: 23 to 28)	0.44 (-1.20, 2.08)	0.597	-1.21, 1.95	0.23 (-0.91, 1.37)	0.690
Attitudes	Age: 41 and above (ref: 23 to 28)	0.25 (-2.45, 2.94)	0.858	-2.57, 2.64	0.54 (-1.05, 2.14)	0.505
Attitudes	Highest pharmacy-related educational qualification: Bachelor (Pharm.D) (ref: Bachelor (B.Pharm))	0.39 (-0.25, 1.03)	0.237	-0.22, 1.01	0.46 (-0.13, 1.05)	0.123
Attitudes	Highest pharmacy-related educational qualification: Master (ref: Bachelor (B.Pharm))	-0.07 (-1.14, 1.00)	0.897	-1.12, 0.92	0.05 (-0.89, 0.99)	0.916
Attitudes	Highest pharmacy-related educational qualification: Doctor of philosophy (PhD) (ref: Bachelor (B.Pharm))	-0.56 (-2.33, 1.20)	0.532	-2.22, 1.10	-1.32 (-2.96, 0.32)	0.114
Attitudes	Current practice setting: Hospital pharmacy (ref: Community pharmacy)	0.01 (-0.59, 0.61)	0.974	-0.55, 0.56	-0.02 (-0.53, 0.49)	0.945
Attitudes	City of current practice: Jeddah (ref: Makkah)	-0.04 (-0.77, 0.69)	0.908	-0.76, 0.66	-0.12 (-0.77, 0.53)	0.722
Attitudes	City of current practice: Taif (ref: Makkah)	0.13 (-0.66, 0.91)	0.753	-0.64, 0.88	0.03 (-0.69, 0.75)	0.930
Attitudes	City of current practice: Madinah (ref: Makkah)	0.55 (-0.24, 1.34)	0.171	-0.24, 1.25	0.73 (0.06, 1.40)	0.033
Attitudes	Years of experience as a licensed pharmacist: 1-5 years (ref: Less than 1 year)	-0.70 (-1.35, -0.05)	0.035	-1.33, -0.04	-0.68 (-1.26, -0.09)	0.023
Attitudes	Years of experience as a licensed pharmacist: 6-10 years (ref: Less than 1 year)	-0.78 (-1.99, 0.43)	0.208	-1.98, 0.39	-0.38 (-1.39, 0.64)	0.464
Attitudes	Years of experience as a licensed pharmacist: More than 10 years (ref: Less than 1 year)	0.05 (-1.45, 1.55)	0.944	-1.47, 1.61	0.65 (-0.49, 1.79)	0.261
Practices	Gender: Female (ref: Male)	-0.97 (-1.96, 0.01)	0.053	-1.90, 0.00	-0.66 (-1.54, 0.21)	0.137
Practices	Age: 29 to 35 (ref: 23 to 28)	0.20 (-1.05, 1.45)	0.754	-1.04, 1.41	0.51 (-0.52, 1.53)	0.332
Practices	Age: 36 to 40 (ref: 23 to 28)	1.60 (-0.76, 3.96)	0.184	-0.57, 4.09	1.74 (-0.20, 3.69)	0.078
Practices	Age: 41 and above (ref: 23 to 28)	1.16 (-2.12, 4.45)	0.487	-1.89, 4.36	0.36 (-1.83, 2.54)	0.748
Practices	Highest pharmacy-related educational qualification: Bachelor (Pharm.D) (ref: Bachelor (B.Pharm))	-0.21 (-1.33, 0.92)	0.716	-1.33, 0.88	0.11 (-0.89, 1.10)	0.834

Outcome	Term	Main: β (95% CI, HC3)	Main p	Bootstrap 95% CI	Excl. influential: β (95% CI)	Excl. influential p
Practices	Highest pharmacy-related educational qualification: Master (ref: Bachelor (B.Pharm))	0.32 (-1.32, 1.96)	0.704	-1.21, 1.97	-0.04 (-1.52, 1.43)	0.953
Practices	Highest pharmacy-related educational qualification: Doctor of philosophy (PhD) (ref: Bachelor (B.Pharm))	-0.85 (-3.00, 1.30)	0.439	-2.77, 1.33	-1.22 (-3.04, 0.60)	0.189
Practices	Current practice setting: Hospital pharmacy (ref: Community pharmacy)	0.05 (-0.88, 0.99)	0.913	-0.84, 0.94	0.26 (-0.53, 1.06)	0.516
Practices	City of current practice: Jeddah (ref: Makkah)	-0.17 (-1.47, 1.14)	0.803	-1.42, 1.14	-0.72 (-1.78, 0.35)	0.186
Practices	City of current practice: Taif (ref: Makkah)	0.58 (-0.69, 1.84)	0.370	-0.65, 1.86	-0.18 (-1.27, 0.92)	0.752
Practices	City of current practice: Madinah (ref: Makkah)	0.37 (-0.98, 1.73)	0.588	-0.98, 1.69	-0.34 (-1.48, 0.80)	0.556
Practices	Years of experience as a licensed pharmacist: 1-5 years (ref: Less than 1 year)	-1.54 (-2.61, -0.47)	0.005	-2.52, -0.50	-1.14 (-2.13, -0.15)	0.025
Practices	Years of experience as a licensed pharmacist: 6-10 years (ref: Less than 1 year)	-1.86 (-3.51, -0.20)	0.028	-3.52, -0.16	-1.34 (-2.78, 0.09)	0.066
Practices	Years of experience as a licensed pharmacist: More than 10 years (ref: Less than 1 year)	-1.41 (-4.08, 1.26)	0.300	-4.05, 1.10	-0.37 (-2.06, 1.32)	0.668

Main analysis: ordinary least squares on all 380 participants with HC3 robust standard errors. Bootstrap: nonparametric case resampling (2000 replicates), percentile 95% CI. Excluding influential observations: model refitted after removing observations with Cook's distance > 4/n (Knowledge: 26; Attitudes: 20; Practices: 19).
